# Incremental Hemodialysis Practices and Impact on Survival: Systematic Review and Meta-Analysis

**DOI:** 10.1016/j.xkme.2025.101238

**Published:** 2026-01-07

**Authors:** Adil M. Hazara, Abdullah Abdullah, Victoria Allgar, Maureen Twiddy, Sunil Bhandari

**Affiliations:** 1 Department of Renal Medicine, York and Scarborough Teaching Hospitals NHS Foundation Trust, United Kingdom; 2 Hull York Medical School/Department of Health Sciences, University of York, Heslington, York, United Kingdom; 3 Blackpool Teaching Hospitals NHS Foundation Trust, United Kingdom; 4 Faculty of Health, Peninsula Medical School, University of Plymouth, Plymouth, United Kingdom; 5 Institute of Clinical and Applied Health Research, University of Hull, Hull, United Kingdom; 6 Hull University Teaching Hospitals, NHS Foundation Trust, Hull, United Kingdom

**Keywords:** Early mortality, end-stage renal disease, incremental hemodialysis, mortality, twice-weekly hemodialysis

## Abstract

**Background:**

Starting hemodialysis (HD) incrementally may help reduce the burden of treatment in patients with established kidney failure. A systematic review has been conducted to describe variations in the practice of incremental HD worldwide and to study its impact on mortality.

**Study Design:**

Systematic review.

**Setting & Population:**

Patients with established kidney failure starting HD.

**Selection Criteria for Studies:**

Medline and Academic Search Premier were searched from inception to July 20, 2020, for observational and interventional studies comparing incremental or twice weekly treatments with conventional treatment, with mortality as one of the outcome measures.

**Predictor:**

Incremental (or twice-weekly) HD therapy versus thrice-weekly treatments.

**Outcomes:**

Mortality.

**Results:**

Fourteen studies were included (combined 91,928 participants; 5,075 [5.5%] in the intervention, 86,853 [94.5%] in standard treatment groups). Large variations in the practice of incremental HD were noted with treatments differing in HD frequency, treatment goals, monitoring schedules, duration of the incremental program, and cointerventions. Larger studies with lowest risk of bias demonstrated equivalent survival between incremental and conventional treatment groups. Meta-analysis of mortality hazards showed an overall hazard ratio of 0.97 (95% CI 0.76-1.19). Centers which screened patients for adequacy of residual kidney function at baseline and pursued prespecified treatment goals have demonstrated better outcomes in incremental HD recipients compared with conventional treatment. There is evidence of publication bias in the literature.

**Limitations:**

Studies from diverse settings, searches limited to English language.

**Conclusions:**

There is wide variation in the interpretation of incremental HD treatments and therefore primary studies of incremental HD must be examined in the context of their settings, population, and available resources. Optimal method of implementing incremental HD remains controversial; available data indicate that incremental HD is at least noninferior to conventional HD.

## Plain-language Summary

The conventional way of starting dialysis in people with kidney failure is to deliver thrice-weekly sessions from the outset. In incremental dialysis, people receive fewer sessions at the start, and their treatment is gradually increased. We have analyzed studies comparing incremental with conventional dialysis treatments. We show that practices vary widely across the world, including how incremental dialysis is given and how patients are monitored. Overall, survival was similar between incremental and conventional programs. Some centers have shown that incremental dialysis may be better when used in carefully selected patients with set treatment goals. However, differences in study quality and reporting make it hard to draw firm conclusions and more research is needed to find the best way forward.

Incremental hemodialysis (HD)–the delivery of shorter and fewer dialysis sessions at the start with gradual increases in therapy as residual kidney function (RKF) declines–seems an intuitive therapeutic option in patients with advanced chronic kidney disease who are about to start long-term HD.^1^^,^^2^ Incremental HD could help reduce the burden of treatment during the early days of dialysis while patients are still adapting to the radical new changes in their daily routines, work commitments, and travel arrangements, as well as to the new pathophysiological changes from being exposed to dialysis therapy.^3^, ^4^, ^5^

There are no full-scale randomized controlled trials (RCTs) comparing incremental and conventional start of HD. In the absence of definitive evidence, guidelines writers and policy makers have so far refrained from making any recommendations on the optimal starting strategy, leaving a large gap in an important area of clinical practice.^6^^,^^7^ Even before the evidence for its efficacy can be examined, it is important to explore the diversity in the practice of incremental HD described in the literature. Because there are no set standards guiding clinical practice, the term incremental HD has been used to describe a diverse set of interventions all converging on the concept of providing dialysis sessions with reduced frequency (usually twice-weekly [2× weekly] treatments), but varying in other fundamental aspects of treatment such as dialysis modality, sessional duration, and progression steps.^8^, ^9^, ^10^

This systematic literature review aims to address unanswered questions on incremental HD in terms of variations in current practices and its impact on early survival.^11^ Our objectives are as follows: (1) to describe various implementations of incremental HD worldwide in terms of characteristics of treatment regimen, setting, and patient population and (2) to study the impact of incremental HD on all-cause mortality.

## Methods

### Search Strategy

Medline and Academic Search Premier were searched from their inception date to 20 July 2020 using a 2-step approach. First, keywords for hemodialysis (hemodialysis OR hemodialysis OR dialysis) were used in the titles and abstracts. Then, keywords were used for incremental or 2× weekly dialysis (incremental OR twice$) in abstracts. Results from these steps were combined with the operator “AND”. The “apply equivalent subjects” qualifier was enabled as an Expander and searches were limited to English language. Studies obtained through hand-searches of reference lists of included articles were added to the final list.

### Eligibility Criteria and Study Selection

Studies were included if they met the following criteria:

1. Original case–control or cohort studies and prospective interventional studies (including RCTs).
2. Involving patients starting or receiving HD or hemodiafiltration (HDF) therapy for established kidney failure in outpatient setting.
3. Must compare incremental HD/HDF (or any regime comprising less that 3× weekly sessions) with conventional treatment defined as HD/HDF sessions given 3× weekly.
4. All-cause mortality is one of the outcome measures.

Titles and abstracts of citations obtained through database searches were screened independently by 2 reviewers. The eligibility criteria were applied independently by two authors on full-text articles of potentially relevant studies with any disagreements resolved through mutual discussion.

### Data Extraction

Basic study identifiers, settings, and participant characteristics from each study were documented on a spreadsheet. Key dialysis treatment characteristics were also extracted including treatment modality (HD or HDF), sessional frequency and duration, clearance of urea achieved, criteria for increment in dialysis treatment, and details of cointerventions. Finally, number of deaths in each group and summary estimates for relative risk or hazard for mortality were recorded. A single author (AMH) extracted data.

There is possible conflation in the literature between the terms 2× weekly and incremental HD.^9^ Although most incremental regimes start with 2× weekly treatments, not all patients receiving 2× weekly HD may be on incremental regimes.^2^ In this systematic review, the term treatment goal has been used to indicate regime type. An overall impression of treatment goal was formed from the study report and using the following principles:

• Goal-directed therapy: the strategy of pursuing prespecified clearance targets and/or maintenance of urine output (or RKF) above a minimum threshold
• Empirical treatment: treating patients based on their symptoms (eg, related to changes in fluid loads, uremic symptoms, or palliative care needs), clinical findings, laboratory results (eg, blood urea levels, potassium levels, hemoglobin levels, phosphate levels, etc.) or personal circumstances (eg, ability to pay, employment, etc.).^12^

Studies that carried out urine output (or RKF) measurements at baseline and then regularly throughout treatment with later increases in dialysis were deemed to have targeted RKF preservation; hence, they were considered goal-directed therapies.

### Risk of Bias Assessment

Risk of bias within studies was assessed using The Risk of Bias In Non-randomized Studies of Interventions (ROBIN-I) tool (Figure S1).^13^ Briefly, this tool contains signaling questions prompting users to rate the quality of studies in 7 separate domains. The overall risk of bias in each study is derived from a judgement of relative impact of biases in each of the above subdomains. Risk of bias assessment was performed independently by 2 authors (AMH and AA), with any differences resolved by mutual agreement.

### Effect Measures

Hazard ratios (HRs), 95% confidence intervals (95% CIs), and *P* values (from Cox analysis or log-rank test) were recorded. When HRs were available from more than 1 model, the output of the maximally adjusted model was used. When HRs and 95% CIs were not reported, methods described in Tierney et al^14^ were used to estimate these from study data. To obtain HR using this method, studies must either report O-E (observed minus expected events) and V (variance) or these must be estimated from summary statistics, including data presented in Kaplan–Meyer curves.^14^

### Synthesis of Results

A narrative synthesis is presented; in the narrative accounts, more weight is given to larger and least biased studies when reporting outcomes. Meta-analyses of HRs have been performed comparing mortality in patients receiving incremental HD versus comparator regime using random effects model with study weights estimated by inverse variance method. Meta-analyses were also performed in subgroup of studies/regimes that pursued a set treatment goal (clearance targets or preservation of urine output) versus those that treated patients empirically. Statistical analyses were completed using Stata/BE 17 (StataCorp LLC, USA).

### Registration

Ethics approval was not required for this type of study. The conduct, analysis, and reporting of this study was supervised by a 5-member Thesis Advisory Panel at Hull York Medical School, the United Kingdom.

## Results

### Study Selection

**Figure 1:**
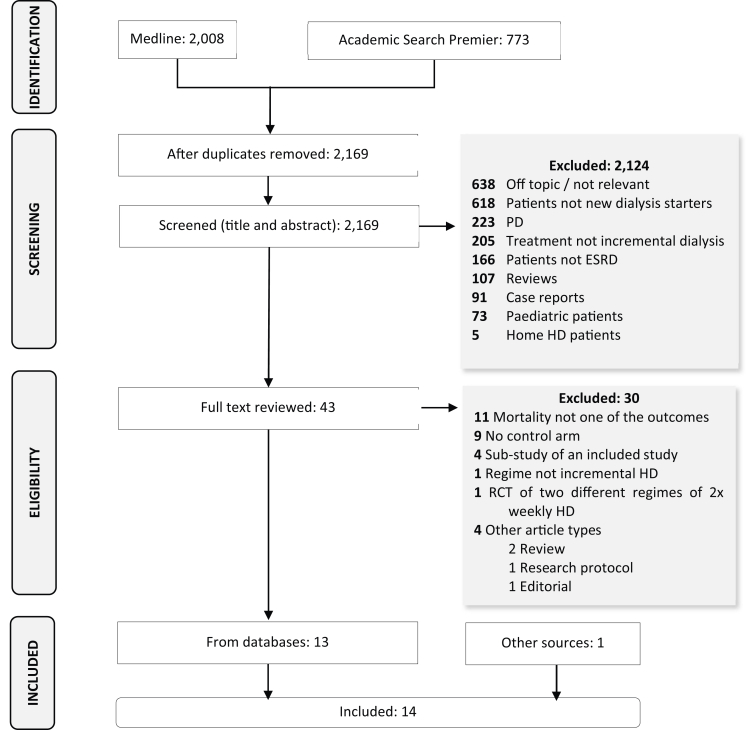
PRISMA flow chart.

In total, 14 studies met the predefined eligibility criteria (Table 1, Fig 1).^15^, ^16^, ^17^, ^18^, ^19^, ^20^, ^21^, ^22^, ^23^, ^24^, ^25^, ^26^, ^27^, ^28^ Together, they represented 91,928 participants receiving long-term HD/HDF. The median was 691 participants, ranging between 68 (Caria et al^18^) and 50,596 (Mathew et al^24^). Eleven were retrospective studies.^15^^,^^16^^,^^19^, ^20^, ^21^, ^22^, ^23^, ^24^^,^^26^, ^27^, ^28^ Three were prospective cohort studies.^17^^,^^18^^,^^25^ With the exception of Hanson et al,^28^ all studies were published after 2010 with the majority (9 out of 14) after 2015 signifying, increasing interest in the subject.

**Table 1:** Studies Included in the Systematic Review.

Author	Treatment Intent for 2x Weekly Treatments	Study Design	Setting - Country	Setting - Center Type	N	n [Intv Group]	n [Cont Group]	Treatment Frequency - Weekly [Intv Group]	Regime [Cont Group]	Mortality Outcome Metric (HR, RR etc.)	Mortality Result	Advantage
Davenport et al^15^ (2019)	Goal directed: to meet minimum clearance target	Retrospective cohort (cluster level comparison)	UK	Multicenter	709	254	455	2x	3x	HR	0.64 (0.43-0.93)	**Intv**
Kaja Kamal et al^16^ (2019)	Goal directed: to meet minimum clearance target	Retrospective cohort	UK	Single center	565	154	411	2x	3x (target total Kt/V of > 1.2 per session)	HR	0.76 (0.57-0.99), *P* = 0.044	**Intv**
Park et al^17^ (2017)	Goal directed: Preservation of RRF	Prospective cohort	Korea	Multicenter, 31 centers participating in the CRC-ESRD study	312	105	207	≤2x	3x	HR	1.10 (0.62-1.97)	**No Diff**
Caria et al^18^ (2014)	Goal directed: Preservation of RRF	Prospective cohort	Italy	Multicenter	68	38	30	1x (with protein restricted diet)	3x	Number of deaths	3/38 in Intv group, 4/30 in control group	**No Diff**
Fernández Lucas et al^19^ (2014)	Goal directed: to meet minimum clearance target	Retrospective cohort	Spain	Single center	134	70	64	2x	3x, (n = 64) – only 31 patients had KRU > 2.5 mL/min	Number of deaths	Number of deaths over follow-up period (log rank χ^2^ - χ^2^ test 0.587, *P* = 0.444)	**No Diff**
Chaker et al^20^ (2020)	Empirical	Retrospective cohort	Tunisia	Single center	88	30	58	1x or 2x weekly	3x	Percentage deaths in 6 months	30% [intv] and 13.8% [Cont], *P* = 0.07	**No Diff**
Wolley et al^21^ (2019)	Empirical	Retrospective cohort	Australia and New Zealand	Multicenter, population wide data from ANZ registry	27,513	850	26,663	<3x at the start, regardless of future changes	≥3x	HR	1.03 (0.92-1.16)	**No Diff**
Yan et al^22^ (2018)	Empirical	Retrospective cohort	China	Multicenter, 45 randomly selected centers participating in DOPPS China study	1,265	256	1,009	2x	3x	HR	1.15 (0.66-2.00) and 1.10 (0.68-1.79) for patients with RRF (n = 413) and those without RRF (n = 852)	**No Diff**
Mukherjee et al^23^ (2017)	Empirical	Retrospective cohort	India	Single center	117	35	82	2x	3x	Number of deaths, mortality rates, mortality rate ratio	Number of deaths: 4 vs 17; Mortality rates: 0.14 vs 0.27 over the entire follow-up; Morality rate ratio: 0.79 (0.40-1.57)	**No Diff**
Mathew et al^24^ (2016)	Empirical	Retrospective cohort	US	Multicenter, 1,737 dialysis facilities run by a private provider	50,596	434	50162	Regular ≤ 2 HD sessions per week in months 4-6 post initiation. (Missing data on CL_urea_ 65%).	3x	HR	0.88 (0.72-1.08)	**No Diff**
Panaput et al^25^ (2014)	Empirical	Prospective cohort	Thailand	Multicenter	673	504	169	2x	3x	HR	HR 0.99 (95% CI 0.47-2.08)	**No Diff**
Lin et al^26^ (2012)	Empirical	Retrospective cohort	China	Multicenter, 58 hospitals in Shanghai	2572	1041	1531	2x	3x	RR	0.77 (0.55-1.09)	**No Diff**
Stankuvienė et al^27^ (2010)	Empirical	Retrospective cohort	Lithuania	Multicenter	2,428	1,008	1,420	1x or 2x	3x	RR	1.92 (1.64-2.24)	**Cont**
Hanson et al^28^ (1999)	Empirical	Retrospective cohort	US	Multicenter, population wide, randomly selected patients from USRDS registry	4,888	296	4,592	2x	3x	RR	0.85, *P* = 0.31 (twice weekly vs standard treatment)	**No Diff**

Abbreviations: ANZ, Australia and New Zealand; Cont, control group; DOPPS, Dialysis Outcomes and Practice Patterns Study; ESRD, end-stage renal disease; HD, haemodialysis/hemodiafiltration; HR, hazard ratio; Intv, intervention group; No Diff, no difference between intervention and control groups; RR, relative risk; RRF, residual renal function; UK, United Kingdom; US, United States; USRDS, United States Renal Data System.

### Participant Characteristics

Of the pooled patient population (n = 91,928), 5,075 (5.5%; mean age 60.7 years) were part of intervention groups (receiving incremental or 2× weekly HD) and 86,853 (94.5%; mean age 60.7 years) were in control groups (receiving 3× weekly HD). The proportion of males was lower in the intervention groups relative to control groups (49% vs 63%, *P* < 0.0001), indicating that women were significantly more likely to receive incremental or 2× weekly HD. Detailed information on comorbid conditions were available in 7 studies (combined number of participants 85,653).^15^^,^^16^^,^^21^, ^22^, ^23^, ^24^^,^^28^ Cardiac (34.7% vs 30.9%) and cerebrovascular disease (14.7% vs 12.2%) were more common in the intervention group, whereas diabetes (28.0 vs 35.6%), peripheral vascular disease (15.0% vs 18.5%), and cancers (20.1% vs 21.3%) were more common in the control groups. Most studies excluded patients receiving dialysis for less than 3 months.

### Risk of Bias Within Studies

The overall risk of bias and risk of bias within the seven ROBIN-I domains for each study are summarized in Figure S2. One study, accounting for 55.0% (50,596 participants) of all participants in the systematic review, was judged to be at low risk; 5 studies (combined participants: 34,543; 37.6%) at moderate risk; 7 studies (combined participants: 6,701; 7.3%) at serious risk; and 1 study (participants: 88; 0.1%) at critical risk of bias.^15^, ^16^, ^17^, ^18^, ^19^, ^20^, ^21^, ^22^, ^23^, ^24^, ^25^, ^26^, ^27^, ^28^

### Regime Characteristics

#### Weekly Treatment Frequency and Duration

In 8 studies, incremental regimes consisted of fixed 2× weekly treatments.^15^^,^^16^^,^^19^^,^^22^^,^^23^^,^^25^^,^^26^^,^^28^ In 4 studies, any treatments delivered at an average of ≤ 3× weekly (including 1× and 2× weekly) were labeled as “incremental” regardless of intent.^17^^,^^20^^,^^21^^,^^27^ In one study, the treatment frequency was 1× weekly.^18^ The control groups all received 3× weekly treatments. The weekly duration of dialysis therapy was not reported in most studies and is presumed to be 12 hours/week. In Hanson et al,^28^ the incremental group received on average 6.2 hours of therapy weekly (vs 12 hours weekly in the control group). This figure was 9.7 hours/week in Lin et al^26^ and 4 hours/week in Caria et al.^18^

#### Achieved Clearances

In studies reporting clearances, median spKt/V_urea_ ranged between 1.4 and 1.7 per session in the intervention groups and between 1.3 and 1.9 per session in the control groups.^15^^,^^17^^,^^19^^,^^21^, ^22^, ^23^, ^24^, ^25^, ^26^ Median eKt/V_urea_ ranged between 1.2 and 1.3 per session in the intervention groups and between 1.1 and 1.5 per session in the control groups.^15^^,^^22^ In populations receiving dialysis at different frequencies (eg, 2× or 3× weekly), clearances may be better expressed as weekly standard “stdKt/V_urea_”. This figure was reported in 4 studies, in which median stdKt/V_urea_ ranged between 1.4 to 3.4 per week in the intervention group and between 2.2 to 5.1 per week in the control groups.^17^^,^^21^^,^^25^^,^^26^

### Treatment Goals

A statement of treatment intent/goal (prespecified goal) was lacking in most studies, except in 5.^15^, ^16^, ^17^, ^18^, ^19^ Kaja Kamal et al^16^ and Davenport et al^15^ both pursued the aim of providing a urea clearance of at least stdKt/V_urea_ ≥ 2.1/week to patients on incremental HD. Fernandez Lucas et al^19^ aimed to escalate treatment frequency if patients’ measured KRU decreased below 2.5 mL/min. Park et al^17^ increased dialysis if urine volume dropped below 200 mL/24 hour. Caria et al^18^ targeted continued maintenance of urine output through regular monitoring of RKF.

### Empirical 2× Weekly HD Therapy

In contrast to the above 5 studies, the treatment intent was not described in 9 studies.^15^, ^16^, ^17^, ^18^, ^19^, ^20^, ^21^, ^22^, ^23^, ^24^, ^25^, ^26^, ^27^, ^28^ These studies were deemed to have treated patients empirically. A common feature within these studies was a lack of regular urine output/RKF measurements in the majority of participants, no prespecified treatment targets, and lack of description of incremental steps.

In 3 of the 9 studies in this category, 2× weekly therapy was the default starting option in the base population, ie, countries where the majority of patients with established kidney failure receive 2× weekly (rather than 3× weekly) therapy.^20^^,^^23^^,^^25^ Therapy regimens in these studies may have followed the imperative of meeting basic patient needs and availability of local funding. For example, in a Thailand based study, government funding only covered 2× weekly treatments, whereas in a study based in India only 9% of patients receiving 2× weekly HD had private health insurance (compared with 31% receiving 3× weekly HD).^23^^,^^25^ Stankuvienė et al^27^ (Lithuania), Lin et al^26^ (China), and Yan et al^22^ (China) were also based in countries where the prevalence of 3× weekly treatments was generally low (68% in Lithuania and 73% in China during the study periods) and out of pocket costs for health care generally relatively high.^29^

In a further 3 studies, participants were selected from large dialysis databases.^21^^,^^24^^,^^28^ Hanson et al^28^ used the United States Renal Data Systems registry. Mathew et al^24^ used databases of a large private provider of dialysis services in the United States, and Wolley et al^21^ used the Australia-New Zealand dialysis and transplant registry. In these retrospective studies, RKF measurements were either not available at all or only available for a minority of patients (in Mathew et al,^24^ 35% of participants had data on RKF measurements at baseline). Key descriptors of treatment regimens including treatment goals, monitoring, and incremental steps were missing from these studies.

### Cointerventions and Duration of Incremental Regime

In Caria et al,^18^ the provision of 2× weekly HD was supplemented by dietary intervention to reduce overall toxin burden. Davenport et al^15^ used bioimpedance analysis in patients receiving 2× weekly HD to assist with assessment of fluid load. Most incremental regimens lasted considerable duration (months and sometimes years) before patients were switched to conventional treatment times (Table 2).

**Table 2:** Duration receiving incremental HD.

Study	At 12 Months	Other Description
Caria et al^18^ (2014)	89% still on inc. HD	-
Park et al^17^ (2017)	77% still on inc. HD	-
Mathew et al^24^ (2016)	68% still on inc. HD	-
Fernández Lucas et al^19^ (2014)	33% still on inc. HD	-
Wolley et al^21^ (2019)	-	Median 8 months
Kaja Kamal et al^16^ (2019)	-	Median 9 months

Presented in descending order (longest to shortest periods). The symbol “–” denotes data not available.

Abbreviation: Inc. HD: incremental haemodialysis.

### Mortality

Of the 14 studies in this systematic review, 11 (combined participants 88,226; 96.0% of total) reported no difference in mortality in patients receiving incremental/2× weekly HD compared with control groups.^17^, ^18^, ^19^, ^20^, ^21^, ^22^, ^23^, ^24^, ^25^, ^26^^,^^28^ Two studies (combined participants 2,428; 2.6% of total) reported improved, and one study (n = 1,274) reported worse survival in patient receiving incremental/2× weekly HD compared with control groups.^17^^,^^21^^,^^22^ After excluding studies with serious or critical risk of bias, the 6 remaining studies (combined participants 85,139) either showed no difference in mortality between treatment groups or an advantage to the incremental dialysis group.^16^^,^^17^^,^^21^^,^^22^^,^^24^^,^^28^

**Figure 2:**
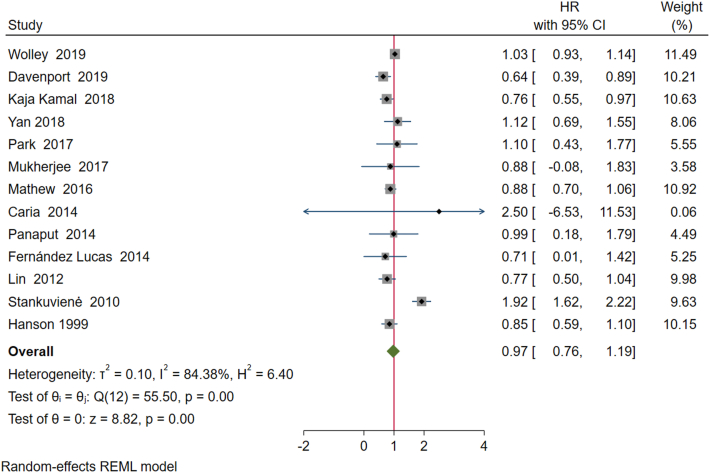
Meta-analysis of all studies.

Meta-analyses have been conducted to support the narrative synthesis. One small study (n = 88, 0.1% of all participants in the systematic review)with critical risk of bias was excluded from meta-analysis.^28^ There was no overall difference in hazard of mortality between those receiving incremental HD versus conventional treatment (HR 0.97, 95% CI 0.76-1.19) (Fig 2). There was evidence of significant statistical heterogeneity as evident by I^2^ statistic of 84.4%; this is further supported by X^2^ test for homogeneity, in which X^2^ of 56 (*P* < 0.05) makes the presence of homogeneity highly unlikely and corroborates the presence of statistical heterogeneity. To further investigate potential source of heterogeneity, a Galbraith plot was drawn (Fig S2). Only one study (Stankuvienė et al^27^) fell outside Galbraith limits; hence, it was the likely source of statistical heterogeneity. When the meta-analysis was repeated following removal of this study, heterogeneity decreased. I^2^ was 35.5%, and an X^2^ of 14 was no longer statistically significant, indicating homogeneity. Repeating meta-analysis after excluding Stankuvienė et al^27^ showed a slight survival advantage associated with incremental HD (HR 0.87, 95% CI 0.76-0.98).

**Figure 3:**
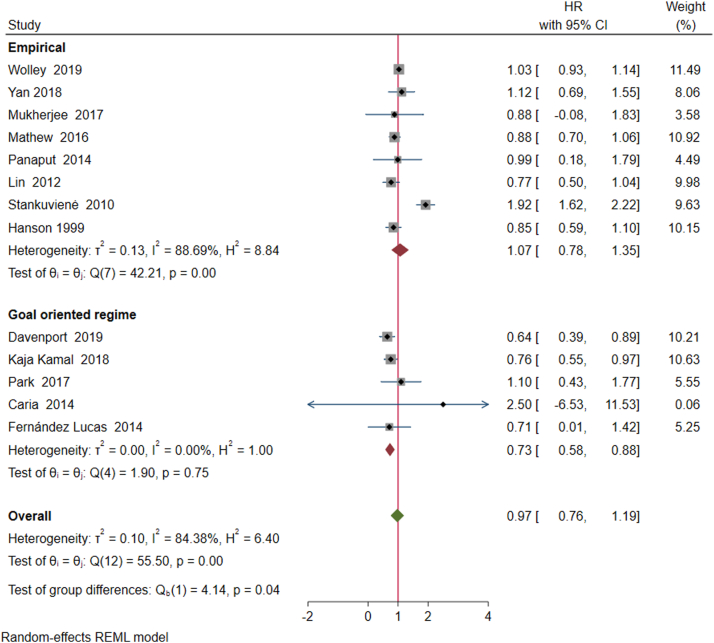
Meta-analysis by studies that chased treatment goals (goal oriented) versus those that did not (empirical). Note that heterogeneity all but disappears when studies that describe goal-oriented regimes are meta-analyzed separately (I^2^ statistic for heterogeneity decreases to 0% in this subgroup of studies).

When meta-analysis was repeated by subgroups based on treatment strategy used within individual studies (empirical treatment vs pursuing a prespecified treatment goal), the hazard of mortality was significantly lower in the latter HR 0.73 (95% CI 0.58-0.88), suggesting that the targeting of prespecified treatment goals may be a superior strategy in implementing incremental dialysis (Fig 3).

**Figure 4:**
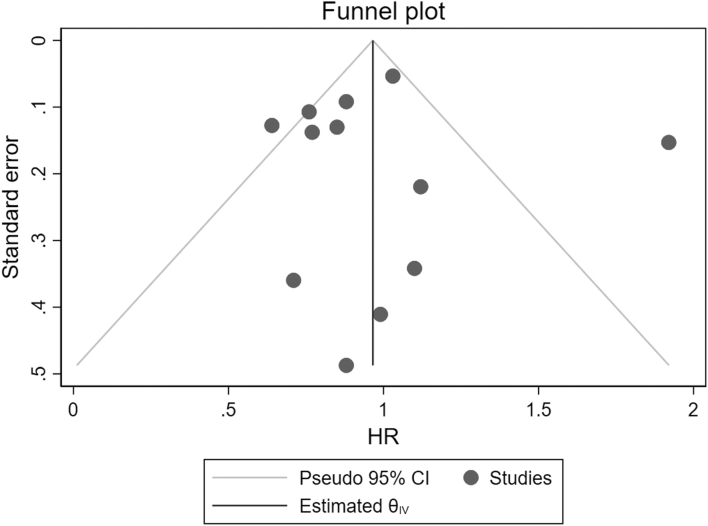
Funnel plot of the studies included in the systematic review; one small study (Caria et al^18^) not shown in the figure because of its very large standard error.

There was possible evidence of reporting/publication bias in favor of incremental HD in this systematic review. The funnel plot in (Fig 4) shows that more precise (larger; smaller standard error) studies were more numerous on the “protective” (HR < 1) side of the funnel.^18^ There were fewer studies on the “harm” (HR > 1) side of the funnel which also tended to be smaller and less precise.

## Discussion

This systematic review aimed to summarize different implementation strategies in the use of Incremental HD worldwide and to study its relationship with survival in patients starting HD. Results show large variations in practice; individual regimens differed significantly in the selection of their patients, settings, treatment goals (or lack of prespecified goals), monitoring schedules, duration of the incremental program, and cointerventions. Incremental HD was associated with similar medium- to long-term survival when compared with standard therapy. There was evidence of publication bias in the reports comparing mortality in incremental HD and standard care recipients.

Previous systematic reviews and meta-analyses comparing outcomes of infrequent with conventional HD therapy have been published.^30^, ^31^, ^32^ Liu et al^30^ report a 20% reduction in mortality and better preserved RKF in association with infrequent HD, whereas Garofalo et al^31^ and Caton et al^32^ found no significant difference in survival between the two treatment groups. In Liu et al,^30^ literature searches included studies up to 2017. In Garofalo et al,^31^ patients receiving both HD and peritoneal dialysis patients were included in mortality analyses.

One key feature that distinguishes our systematic review from previous works is the attention we pay to the type of regime when considering “incremental HD”.^30^, ^31^, ^32^ As highlighted previously, it is important to distinguish between recipients of incremental HD and those receiving 2× weekly HD for other reasons, such as socioeconomics, palliative, and adherence.^33^ Given the retrospective nature of most of studies, determining exposure to true incremental HD (as opposed to receiving 2× weekly HD for other reasons) can lead to bias. Our study treats these separately and hence represents a novel contribution to the literature.

HD therapy at reduced frequency (eg, 1× or 2× weekly) is routinely offered to large number of patients around the world. It is an important means of ensuring that this life-saving therapy reaches a wider subsection of the population when resources are limited.^34^ In the literature, 2× weekly and incremental HD have often been conceptualized together and considered as being interchangeable; many studies labeling any patients receiving less than 3× weekly HD for a continuous period as having received incremental HD. This is particularly the case in studies derived from large retrospective datasets. Analyzing the outcomes for those receiving incremental and 2× weekly treatments as if they were the same entity would clearly lead to false assertions. One of the key strengths of our systematic review is that we have shown sensitivity to such issues when preparing our analyses and in the presentation of data.

Surprisingly, the impact of incremental HD on early mortality, defined as deaths within the first 6 months, remains unexplored. Studies of incremental HD have either excluded patients who were receiving dialysis for less than a certain minimum period (usually less than 90 days) or discounted the outcome “early deaths” from their analyses. The systematic elimination of early mortality counts from these studies has resulted in a large knowledge gap. This needs to be addressed urgently given the potential advantages that incremental HD could offer in improving early outcomes.^35^

Studies brought together in systematic reviews inevitably differ from each other.^32^ The prespecification of PICOTS (population, intervention, comparator, outcome, timing, setting) elements and screening of the studies by 2 authors (AMH, AA) ensured that only clinically and methodologically similar studies were included in the systematic review. In relation to statistical heterogeneity, only 1 study deviated significantly in its effect size expected for the population (as explored using Galbraith plot).^27^ Although we were able to narrow down the source of heterogeneity to study level, it is unclear which characteristic(s) within studies contributed most to heterogeneity. In theory, meta-regression analyses could be used to further explore this; however, this would have required a relatively uniform and complete reporting of data, which was not available in this study (primary authors were not contacted for more data).^36^

Two strategies for implementing incremental HD showed particularly improved outcomes: the selection of patients by the presence of RKF at baseline and the presence of prespecified treatment goals. Note that this was driven by 2 studies in this systematic review from the United Kingdom.^15^^,^^16^ Authors from this center report practicing their version of incremental HD for over 20 years.^16^ The distinctive trait of their regimen is the regular measurements of RKF (through repeated urine collections) and the increase in HD time/frequency with declining RKF; our systematic review supports this strategy. The limitations of this systematic review include the heterogeneity of its constituent studies in terms of their design, setting, and the characteristics of the patient population that they represent. This is an important consideration given the known large variations in practice of hemodialysis world-wide which affect patient survival.^37^ We were also limited by the availability of data in each study, in particular the regime descriptions. Hence, certain decisions such as whether regimes were “goal directed” or “empirical” were based on subjective judgement. We were unable to separate out recipients of HD and HDF from regime description within primary studies. A further limitation is that data extraction was carried out by a single author; international guidelines recommend that at least 2 authors independently perform this task to minimize the impact of subjectivity in interpretation of outcomes.^38^ However, our data and analyses was reviewed regularly by a 5-member Thesis Advisory Panel at the author’s host institution for quality. The strengths of this study are its novelty, the descriptions of variations in practice that aim to be clinically relevant and informative for day-to-day practice, and the new analyses which elucidate the optimal strategy in the practice of incremental HD.

In new HD starters who have substantial RKF and are well prepared for HD (such as those previously seen at predialysis clinics), an incremental HD regime that prespecifies treatment targets or defines incremental steps at the outset appears feasible from these findings. The aim of such a regime should be to ease patients into regular HD therapy over a period of few months.^39^ If the requirement for regular urine collections could be bypassed during safety and adequacy monitoring conducted using conventional means (eg, through measurements of blood biochemistry and body fluid load), this could make incremental HD accessible to higher proportion of patients with kidney failure worldwide.

## Figures and Tables

**Supplementary Figure S1:**
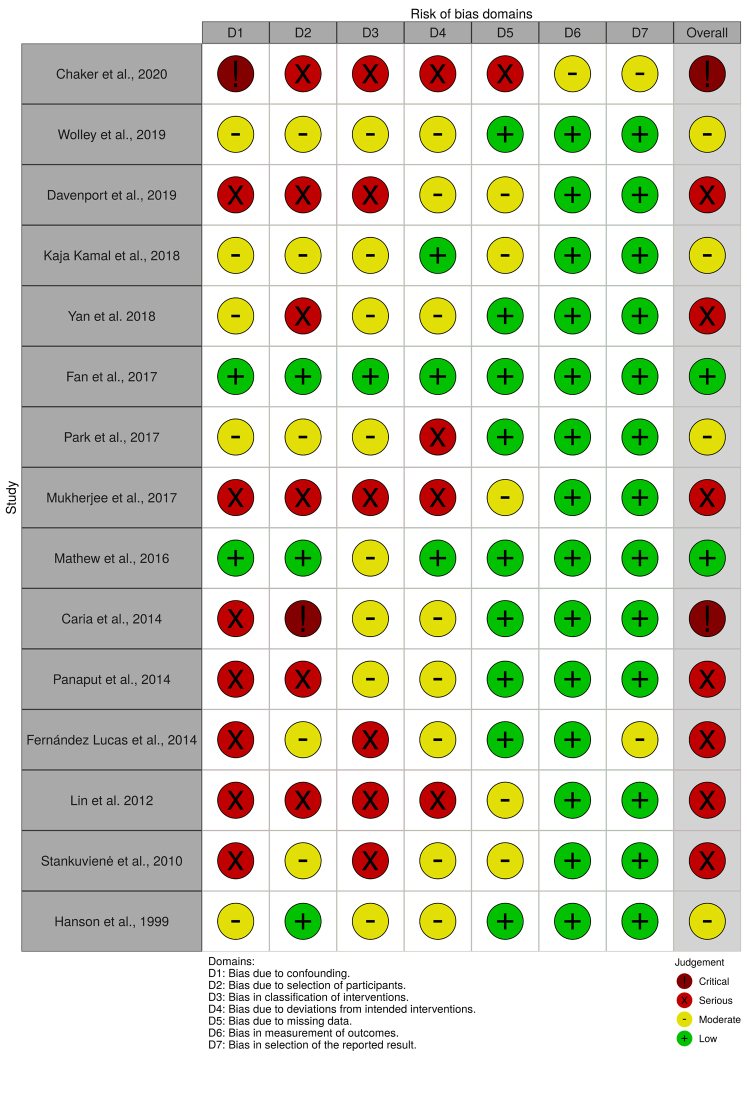
Summary of risk of bias in the studies included in the systematic review. Domains 1 to 7 are those specified in the ROBIN-I tool.

**Supplementary Figure S1:**
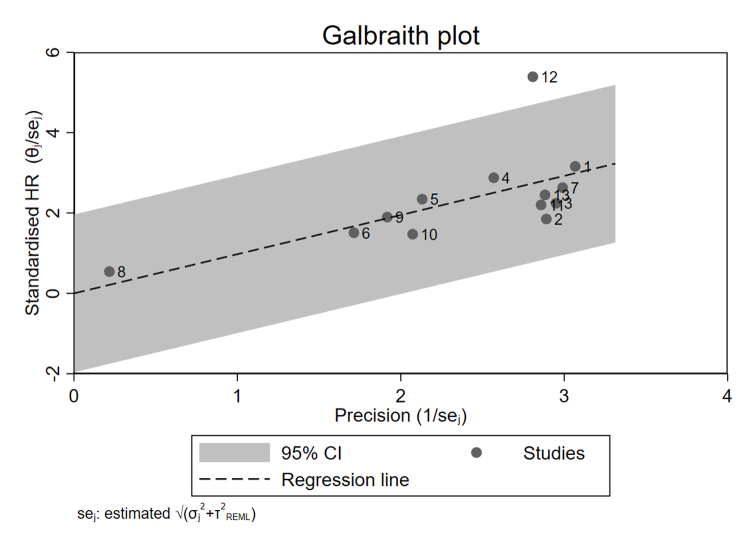
Galbraith plot. Studies are identified by labels 1 to 13. Study 12 represents Stankuvienė et al (2010).^27^
