# Supplementary material for: Incremental Hemodialysis Practices and Impact on Survival: Systematic Review and Meta-Analysis
Source: Kidney Med. 2026 Jan 7;8(3):101238. doi: 10.1016/j.xkme.2025.101238 (PMC12937166; doi:10.1016/j.xkme.2025.101238)
Supplement: Supplementary Table S1 [file mmc1.pdf]

Table S1. PICOS elements used to devise search strategy and eligibility criteria for studies.

Element	Description
P – Participants	Patients with CKD-5 starting or receiving maintenance HD therapy or end-stage renal disease<br><br>Include all: regardless of background of previous specialist follow-up for a minimum of 3 months, ability to provide 24-hour urine sample, patient's socioeconomic status (and medical insurance status) and employment status.
I – Intervention/Exposure	Less than three times (once or twice weekly HD) as the starting regime:<br><br>Include all regardless of additional treatment components (co-interventions) including the monitoring of RRF and adjustments to dialysis prescription based on this, monitoring regime (frequency of clinical reviews, the use of bio-impedance), support with other forms of RRT e.g. PD, dietary and fluid restrictions and whether the intervention is government funded or privately financed.
C – Comparators	Any other HD or HDF based regimes<br><br>Include all: three times weekly HD/HDF, other types of twice weekly HD/HDF
O – Outcomes	Primary: All-cause mortality (or survival)
S – Settings	In-centre out-patient HD/HDF (not home HD, not acute starters in hospital)<br><br>Funding of dialysis services<br><br>• Where provision of HD is fully government funded from the outset<br>• Where there is no government funding and patients are expected to pay full costs (either out of pocket or through private health insurance)<br>• Intermediate (a combination of above – i.e. where there is partly government funded and partly patient funded)<br><br>Patient access to fall back services: is may be a function of overall function of a countries health system classification (OECD)
